# Supplementary material for: Metabonomics and Transcriptomics Analyses Reveal the Underlying HPA-Axis-Related Mechanisms of Lethality in Larimichthys polyactis Exposed to Underwater Noise Pollution
Source: Int J Mol Sci. 2024 Nov 24;25(23):12610. doi: 10.3390/ijms252312610 (PMC11641136; doi:10.3390/ijms252312610)
Supplement: Supplementary file 1 [file ijms-25-12610-s001.zip › Supplementary material legend.pdf]

Supplementary material information:

Figure S1. The underwater sound acquisition system, including UW30 amplifier, audio data collector, HTD42 hydrophone, and the operational software.

Figure S2. All DMs are mapped to the Human Metabolome Database (HMDB). (A) DMs of brain group mapped to the HMDB; (B) DMs of heart group mapped to the HMDB; (C) DMs of adrenal gland group mapped to the HMDB.

Table S1. The RNA sequencing statistics for 18 RNA libraries of three tissues.

Table S2. The expression difference statistics of DEGs in three tissues.

Table S3. The statistical information of DMs.

Table S4. The primer information of qRT-PCR validation.
