# Supplementary figures and images for: Metabonomics and Transcriptomics Analyses Reveal the Underlying HPA-Axis-Related Mechanisms of Lethality in Larimichthys polyactis Exposed to Underwater Noise Pollution
Source: Int J Mol Sci. 2024 Nov 24;25(23):12610. doi: 10.3390/ijms252312610 (PMC11641136; doi:10.3390/ijms252312610)

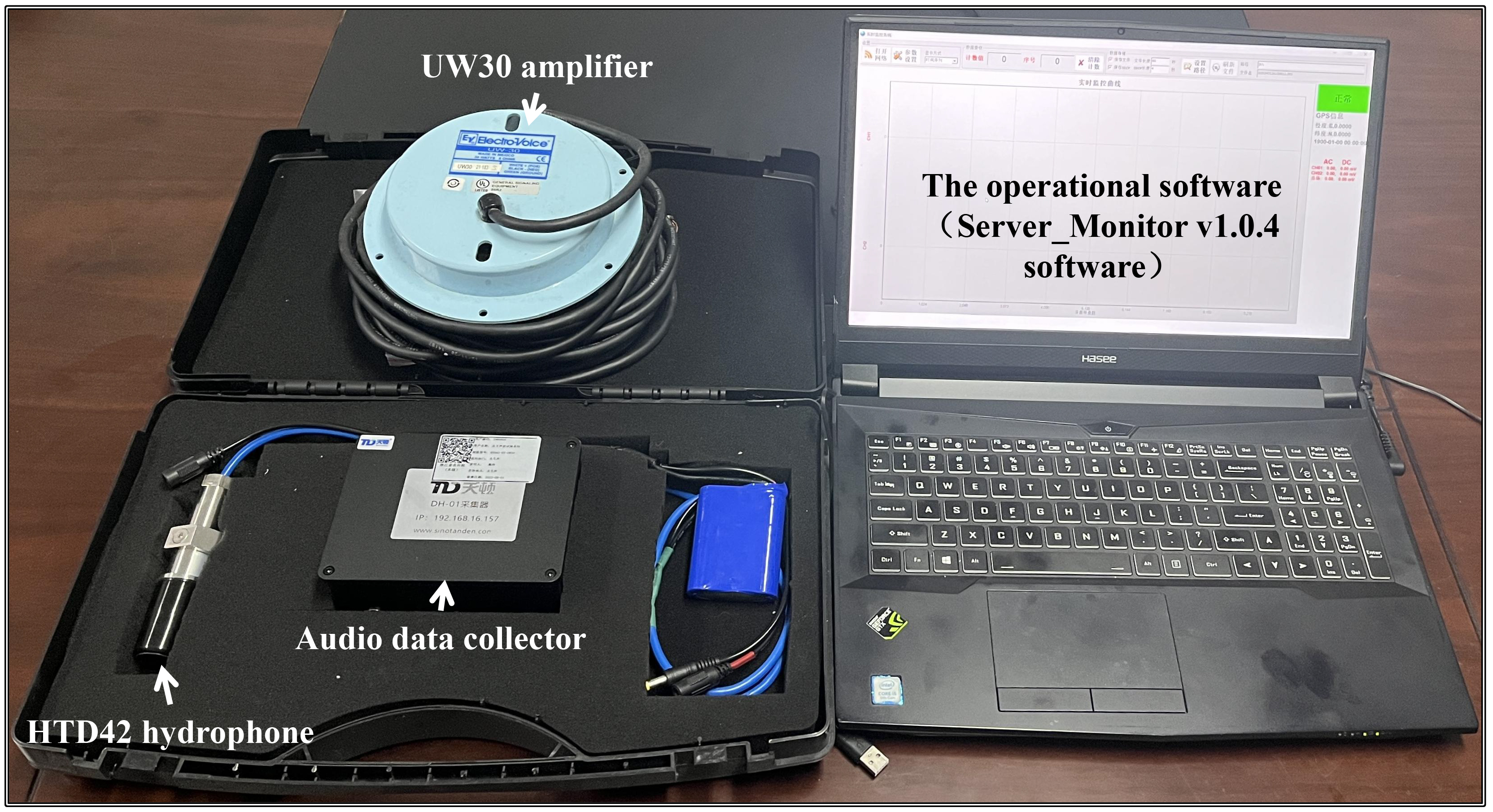

Supplement: Supplementary file 1 [file ijms-25-12610-s001.zip › Supplementary material Figure S1.jpg]

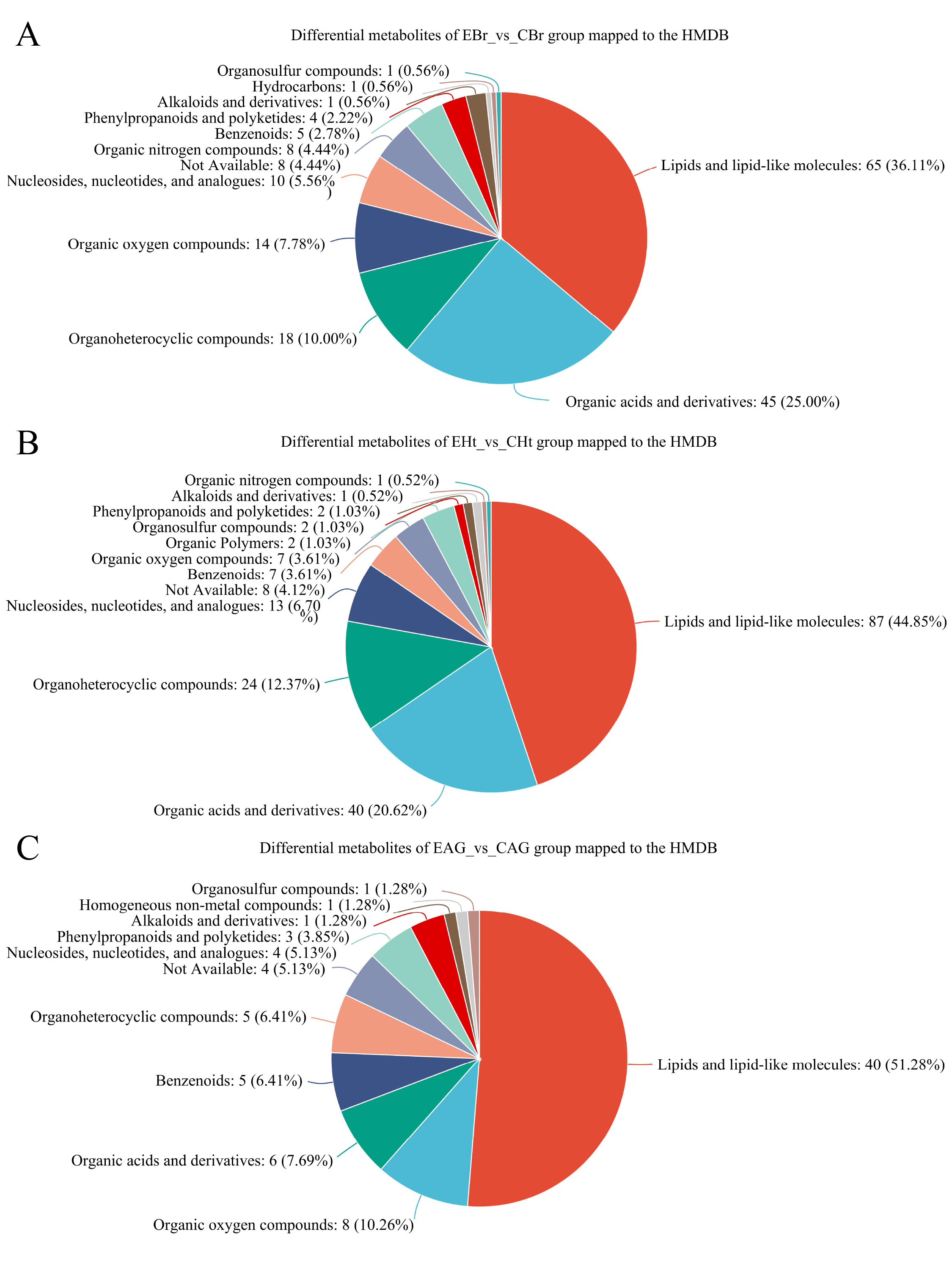

Supplement: Supplementary file 1 [file ijms-25-12610-s001.zip › Supplementary material Figure S2.jpg]
